# Evolution of Electronic State and Properties of Silver Nanoparticles during Their Formation in Aqueous Solution

**DOI:** 10.3390/ijms221910673

**Published:** 2021-10-01

**Authors:** Vadim Ershov, Natalia Tarasova, Boris Ershov

**Affiliations:** 1 Frumkin Institute of Physical Chemistry and Electrochemistry, Russian Academy of Sciences, 119071 Moscow, Russia; vadersh@yandex.ru; 2 Institute of Chemistry and Problems of Sustainable Development, Dmitry Mendeleev University of Chemical Technology of Russia, 125047 Moscow, Russia; tarasnp@muctr.ru

**Keywords:** localized surface plasmon resonance, Mie-Drude theory, silver nanoparticles, electron density, surface, blue shift

## Abstract

The electron density of a nanoparticle is a very important characteristic of the properties of a material. This paper describes the formation of silver nanoparticles (NPs) and the variation in the electronic state of an NP’s surface upon the reduction in Ag^+^ ions with oxalate ions, induced by UV irradiation. The calculations were based on optical spectrophotometry data. The NPs were characterized using Transmission electron microscopy and Dynamic light scattering. As ~10 nm nanoparticles are formed, the localized surface plasmon resonance (LSPR) band increases in intensity, decreases in width, and shifts to the UV region from 402 to 383 nm. The interband transitions (IBT) band (≤250 nm) increases in intensity, with the band shape and position remaining unchanged. The change in the shape and position of the LSPR band of silver nanoparticles in the course of their formation is attributable to an increasing concentration of free electrons in the particles as a result of a reduction in Ag^+^ ions on the surface and electron injection by ${\mathrm{CO}}_{2}^{-}$ radicals. The ζ-potential of colloids increases with an increase in electron density in silver nuclei. A quantitative relationship between this shift and electron density on the surface was derived on the basis of the Mie–Drude theory. The observed blue shift (19 nm) corresponds to an approximately 10% increase in the concentration of electrons in silver nanoparticles.

## 1. Introduction

Nanoparticles have unique properties and are widely used in catalysis [1,2,3,4,5], bioimaging and sensing [6,7,8], diagnostics, and therapies [9,10]. The materials with a Ag coating demonstrate strong antibacterial and antiviral abilities, and potentially can be used to fight COVID-19 [11,12]. The specific optical properties of silver and gold NPs, due to the localized surface plasmon resonance (LSPR), make it possible to use NPs of these metals for the detection of SARS-CoV-2 [13] and MERS-CoV [14].

Colloidal solutions of metals (in the case of aqueous solutions andhydrosols) absorb light in the UV and visible regions. The absorption is due to the excitation of the surface plasmons of ultrafine metal particles and to IBT [15,16,17,18]. The metal nanostructures LSPR can be employed to enhance the light absorption of semiconductor materials, resulting in significantly enhanced photoactivity [19]. The absorption of the LSPR for silver occurs at 380–410 nm, while the lower boundary of the 4 d → 5 sp IBT is approximately 320 nm and the band extends to shorter wavelengths. The absorption of surface plasmons is very sensitive to the state of the silver nanoparticle surface. The particles have a very large, specific surface area, and unsaturated silver adatoms are able to adsorb molecules and ions. Spectrophotometric analysis provides useful information on the state of a particle and its stabilizing layer, sorption of donor and acceptor molecules, and other factors that change the electronic state of the particle surface. The adsorption of molecules and ions changes the density of the conduction electrons in the metal, which induces a shift in the absorption band. The adsorption of donor molecules and ions shifts the LSPR band of silver and gold to shorter wavelengths and, conversely, adsorption of acceptor molecules and ions causes a shift to longer wavelengths [20,21,22,23]. The characteristics of Ag materials shows a noticeable improvement upon the photoexcitation of plasmons in a metal. The luminescence intensity of carbon dots can be improved 5–6 times by LSPR of Ag@SiO_2_ NPs [24]. The photoresponse of the Ag-WS_2_/Si heterostructure device is enhanced due to plasmonic improvement [25]. The question of size-dependent plasmonic resonances in NPs has been investigated and discussed for several decades [16,17,18]. New interest has recently been gained with the introduction of improved experimental techniques allowing for single-particle experiments [26,27].

The inclusion of nanosilver in inorganic heterostructures gives them new, useful properties. For example, hybrid photocatalysts for the formation of H_2_ have been synthesized [28]; an electrocatalyst has been developed in the form of nanosilver deposited on the surface of MoS_2_, which demonstrates a high oxygen reduction reactivity of oxygen [29], and a silver composite on carbon nanotubes showed a high activity of decomposition of toxic dyes and inactivation of bacteria [30].

Unlike the LSPR, which is caused by the presence of free conduction electrons in the metal, the IBT are caused by excitation of the inner valence electrons of the metal (4 d → 5 sp) [17,18,19]. Weak external impacts related to chemisorption and other surface processes do not noticeably affect the IBT absorption, as opposed to the LSPR absorption. IBT absorption is proportional to the concentration of atoms in the silver nanoparticles of smaller sizes.

It may be assumed that the significant difference between the natures of LSPR and IBT bands could be utilized to elucidate the changes in the electronic state and properties of silver nanoparticles during their formation, and to determine the mechanism of metal nucleation in a homogeneous medium. In our previous study, we found that the LSPR absorption band of silver nanoparticles formed during the photoreduction in Ag^+^ ions by oxalate ions gradually shifts to shorter wavelengths [31]. Here, we report the results of a systematic study of this phenomenon, which confirms that it is caused by a change in the solution composition, structure of the electrical double layer, and electronic state of nanoparticles.

## 2. Results and Discussion

Ultraviolet irradiation of a deaerated solution of Ag^+^ ions (1–3 × 10^−4^ mol L^−1^) containing oxalate ions (2–5 × 10^−4^ mol L^−1^) results in the formation of a stable silver hydrosol composed of nano-sized metal particles. Their formation is initiated by the photochemical decomposition of oxalate ions and the formation of ${\mathrm{CO}}_{2}^{-\cdot }$ radical ions [32,33], which reduce Ag^+^ ions to Ag^0^ atoms.

**Figure 1:**
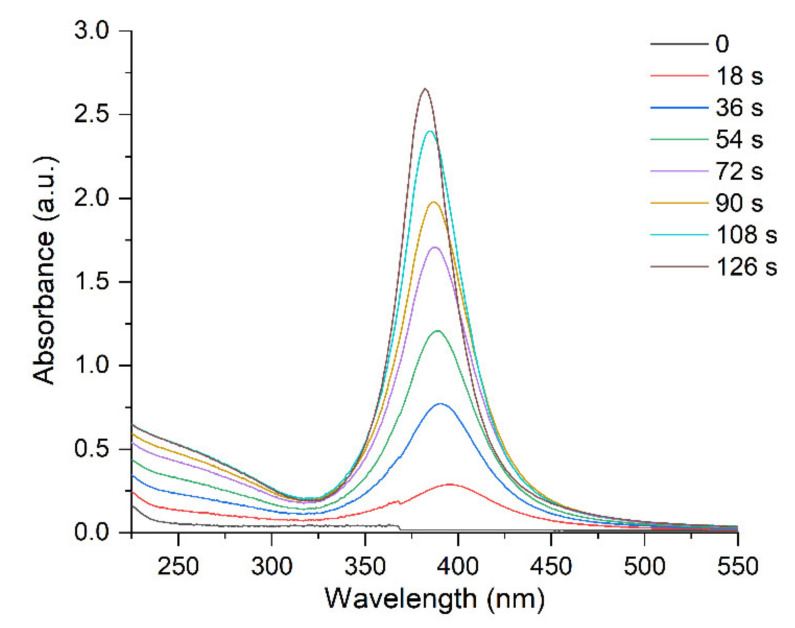
Variation of absorption of the silver hydrosol during its formation under UV irradiation. Solution: Ag^+^ (3 × 10^−4^ mol L^−1^) and $C_{2}O_{4}^{2-}$ (5 × 10^−4^ mol L^−1^). Optical length is 5 mm.

**Figure 2:**
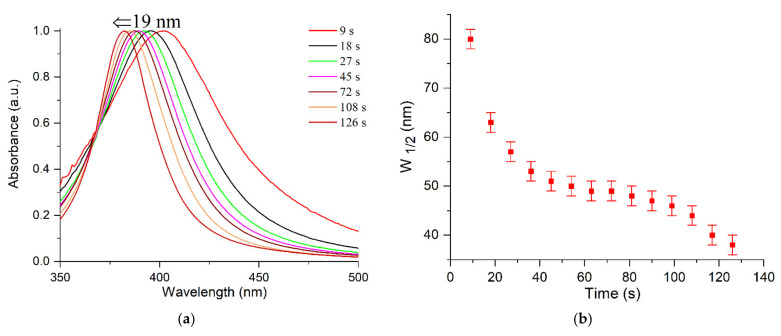

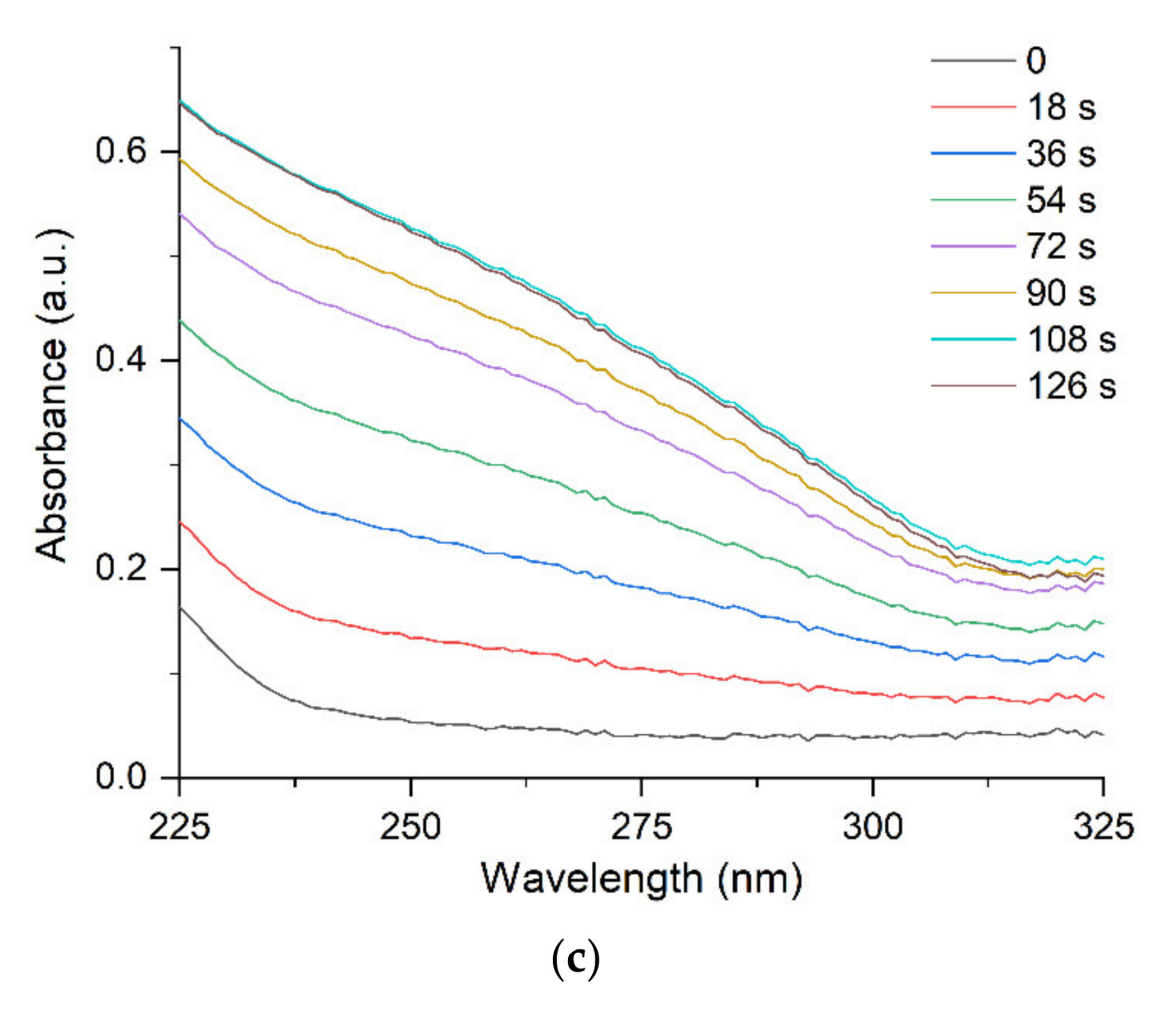
Variation of absorption of the silver hydrosol during its formation under UV irradiation: (**a**) shift of the LSPR band; (**b**) change in the width of the LSPR band; (**c**) change of IBT absorption. Solution: Ag^+^ (3 × 10^−4^ mol L^−1^) and $C_{2}O_{4}^{2-}$ (5 × 10^−4^ mol L^−1^). Optical length is 5 mm.

Figure 1 and Figure 2 illustrate the variation in the optical spectrum of a solution containing Ag^+^ and $C_{2}O_{4}^{2-}$ ions with the time of exposure to the pulsed UV light. UV irradiation was carried out in an evacuated quartz cell with a xenon lamp for a specified time (for example, 9 s), then, the optical spectrum was measured for approximately 1 min. The spectrum did not change in the repeated measurements. The next UV irradiation was then performed, and so on. The times of successive irradiations were summed up. It can be seen that the LSPR band with a maximum at ~400 nm and the IBT band, inherent in silver nanoparticles, appear and increase in intensity with increases in the time of photochemical exposure (Figure 1). The LSPR band gradually shifts to shorter wavelengths (blue shift) and narrows down (Figure 2a,b). The intensity of the LSPR absorption band reached a constant limit value after about 120 s of exposure to UV light. The blue shift of the LSPR band is approximately 19 nm (from 402 to 383 nm) and the band width decreases almost by half from approximately 80 nm to 40 nm).

**Figure 3:**
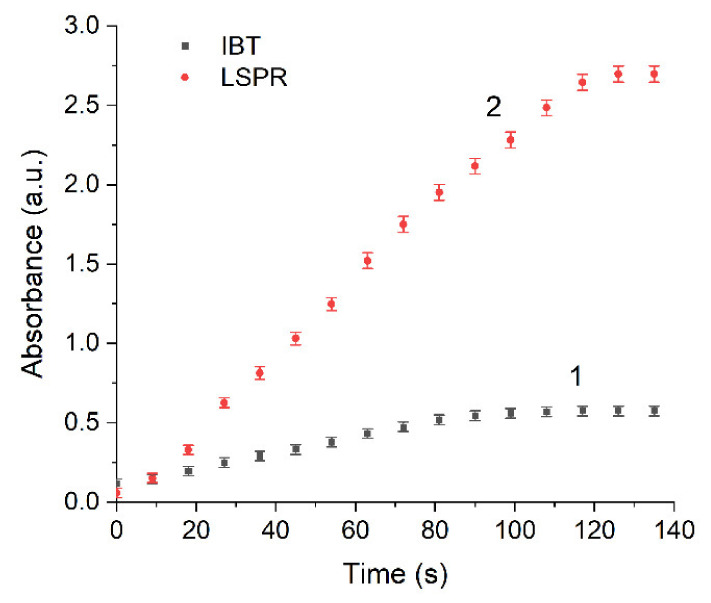
Variation of IBT absorption at 250 nm (curve 1) and LSPR absorption at λ_max_ (curve 2) as functions of the UV irradiation time. Solution: Ag^+^ (3 × 10^−4^ mol L^−1^) and $C_{2}O_{4}^{2-}$ (5 × 10^−4^ mol L^−1^).

Absorption caused by interband transitions of electrons is detected over a broad range from 200 nm to approximately 325 nm, with a diffuse maximum at approximately (255 ± 5) nm. It can be seen (Figure 2c) that IBT absorption increases with increasing time of UV irradiation but, as opposed to LSPR, the band position and shape do not change. The hydrosol formation is completed after approximately 80 s of irradiation. This is manifested as a constant and stable IBT absorption, indicating that all Ag^+^ ions in the solution have been reduced (Figure 3, curve 1). Meanwhile, the LSPR band continues to increase in intensity (Figure 3, curve 2), shift to shorter wavelengths (Figure 2a), and narrow down (Figure 2b) upon further UV irradiation. After approximately 120 s, as mentioned previously, an ultimate stationary absorption of the LSPR band is attained.

**Figure 4:**
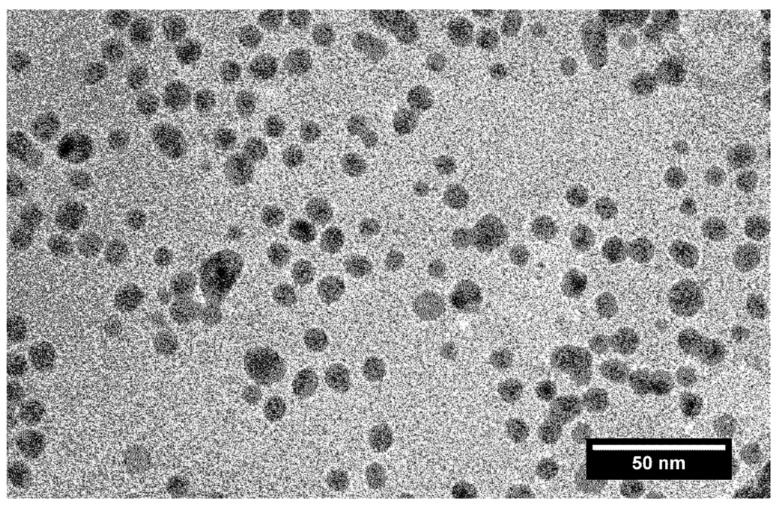
TEM image of silver nanoparticles. Solution: Ag^+^ (3 × 10^−4^ mol L^−1^) and $C_{2}O_{4}^{2-}$ (5 × 10^−4^ mol L^−1^).

The blue shift of the LSPR band indicates that during the reduction of Ag^+^ ions the electron density in the nanoparticles increases. A decrease in the concentration of Ag^+^ ions in a solution upon their reduction leads to a decrease in the number of ions on the surface of nanoparticles, which increases the shift of the LSPR band. The state of the surface and adsorption on the surface do not affect the energy of optical transitions of the inner valence electrons of the metal. The increase in the IBT absorption with increasing UV exposure time reflects the increasing number of silver atoms. A comparison of the plots for the IBT and LSPR absorption bands versus the UV irradiation time indicates that the shift of the LSPR band is not due to the reduction in Ag^+^ ions only. The TEM data indicate that the photochemical reduction in Ag^+^ ions resulted in the formation of spherical silver nanoparticles (Figure 4). The average particle size in a solution containing 3 × 10^−4^ mol L^−1^ of Ag^+^ ions and 5 × 10^−4^ mol L^−1^ of $C_{2}O_{4}^{2-}$ ions is 10.4 ± 3.2 nm. It is noteworthy that silver nanoparticles of approximately the same size (10.1 ± 3.8 nm) were obtained in a solution with a lower Ag^+^ concentration (1 × 10^−4^ mol L^−1^).

**Figure 5:**
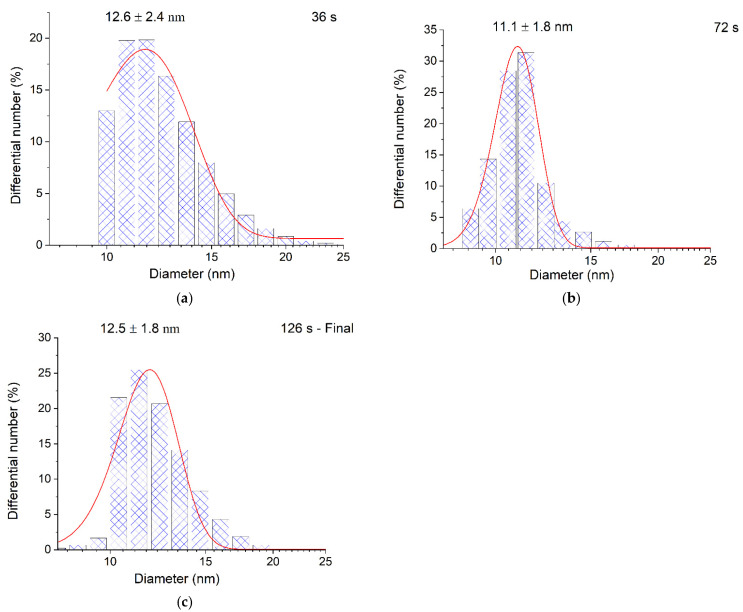
DLS diagrams of particle size distribution during the photochemical reduction of silver in a solution containing 3 × 10^−4^ mol L^−1^ Ag^+^ and 5 × 10^−4^ mol L^−1^${\;C}_{2}O_{4}^{2-}$: (**a**) 36 s; (**b**) 72 s; (**c**) 126 s.

The particle size distribution was also identical. The measurement of particle size by TEM is known to include opening deaerated hydrosol in air, deposition of the hydrosol on a plate, and other procedures. This does not exclude the possibility of a change in the state of hydrosol nanoparticles due to oxidation and aggregation. Therefore, we also used the DLS method, in which the micelle size was measured in a quartz optical cell immediately after preparation of the hydrosol without opening. Figure 5 shows the DLS size distribution curves for the intermediate stages of the photochemical formation of the hydrosol. During 36 s and 72 s of UV irradiation approximately 38% and 78% of Ag^+^ ions are reduced in a 3 × 10^−4^ mol L^−1^ solution, and during 126 s of UV irradiation the ions are completely reduced (Figure 3, curve 1).

It can be seen that the average micelle size measured by DLS in any stage of hydrosol formation is the same within the error and amounts to approximately 11–13 nm. This indicates that in each particular stage of Ag^+^ photoreduction, the resulting silver atoms are aggregated into particles of approximately the same size. Apparently, this is due to the fact that the ${\mathrm{CO}}_{2}^{-\cdot }$ radical ions reduce Ag^+^ to Ag^0^ in the solution bulk, which is favored by the relationship of the potentials: E^0^$\left(\frac{{\mathrm{CO}}_{2}}{{\mathrm{CO}}_{2}^{-\cdot }}\right)$ = –1.9 V [34] and E^0^$(\frac{{\mathrm{Ag}}^{+}}{{\mathrm{Ag}}^{0}})\;$= –1.8 V [35]. Subsequently, short-lived silver clusters (${\mathrm{Ag}}_{2}^{+}$, ${\mathrm{Ag}}_{3}^{2+}$, ${\mathrm{Ag}}_{4}^{2+}$, and ${\mathrm{Ag}}_{8}^{2+}$, etc.) appear [1,2,3,36,37] and agglomerate, finally yielding metal nanoparticles. The process can be conventionally represented by the following equations:

(1) $${\mathrm{Ag}}^{+}+{\mathrm{CO}}_{2}^{-\cdot }\to {\mathrm{Ag}}^{0}+{\mathrm{CO}}_{2}$$

(2) $${\mathrm{nAg}}^{0}\;\to \;{\mathrm{Ag}}_{n}$$

(3) $${\mathrm{CO}}_{2}+H_{2}O\;↔{\;H}^{+}+{\mathrm{HCO}}_{3}^{-}\;↔\;2H^{+}+{\mathrm{CO}}_{3}^{2-}$$

Reaction (1) was studied by a pulse radiolysis method [38] and its rate constant was very high (*k* = 4 × 10^9^ mol L^−1^ s^−1^). The CO_2_ molecules were hydrated and equilibriums (3) were established, giving rise to ${\mathrm{HCO}}_{3}^{-}$ and ${\mathrm{CO}}_{3}^{2-}$ ions. These ions were adsorbed on the surface of silver nanoparticles, and the electrical double layer thus formed ensures the electrostatic stabilization of the metal hydrosol [37]. The subsequent cluster agglomeration proceeds independently of the existing metal phase and ends in the formation of nanoparticles [39]. Apparently, pulsed UV irradiation mainly increases the number of particles in the solution bulk, but has little effect on the particle size. This distinguishes the photochemical method for the generation of silver particles from other methods that use reagents with a lower reduction potential compared to that of the ${\mathrm{CO}}_{2}^{-\cdot }$ radical anion. This results in an autocatalytic mechanism of Ag^+^ reduction on the surface of arising NPs, which leads to increasing nanoparticle size.

**Figure 6:**
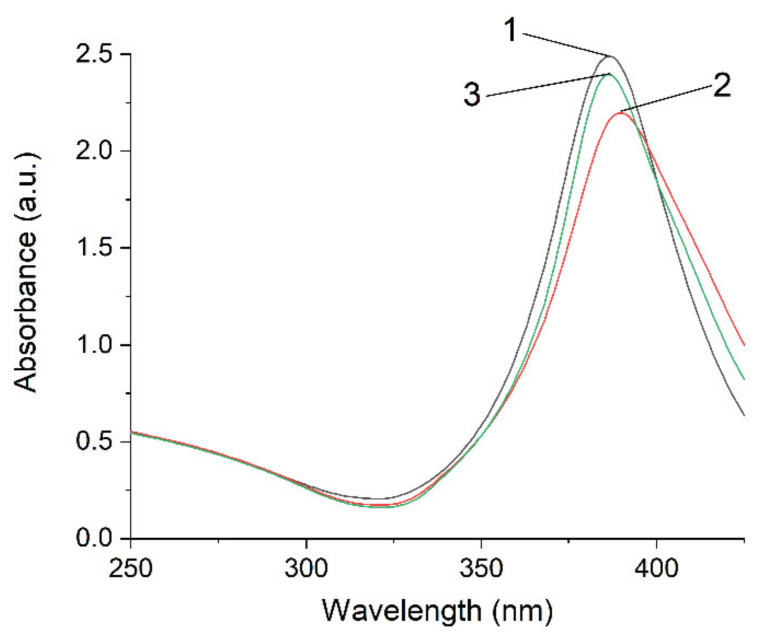
Variation in absorption of the silver hydrosol during its «charging 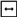 discharging» procedure: 1—after formation; 2—after aging for 3 days; 3—after UV irradiation for 20 s. Solution: Ag^+^ (3 × 10^−4^ mol L^−1^) and $C_{2}O_{4}^{2-}$ (5 × 10^−4^ mol L^−1^). Optical length is 5 mm.

Plasmon absorption is sensitive to the state of the surface. The change in the shape and position of the LSPR band reflects the change in the electronic state of silver nanoparticles during their formation via reduction in Ag^+^ ions. According to the Mie–Drude theory [15,16,17,18], an increase in the electron density on the nanoparticle surface induces a shift of the LSPR band to shorter wavelengths (blue shift), increase in the band intensity, and broadening of the band. These changes in the optical absorption of silver nanoparticles observed in experiments would be reasonably attributed to evolution of the nanoparticle electronic state and properties during their photochemical formation. Nevertheless, the possibility that the changes are caused by other factors cannot be ruled out either. A possible cause is that, during the photochemical generation of nanoparticles, they are rearranged to a more symmetrical geometry, the size distribution is narrowed, and plasmon blue shift takes place. Other causes are also possible. Therefore, we performed experiments to elucidate the influence of charging 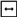 discharging of the hydrosol of a constant composition on the structure of its optical absorption. For this purpose, charged hydrosol (corresponding to absorption after 126 s of UV irradiation in Figure 1) was kept in the dark at a constant room temperature for a long period in which spontaneous discharging took place. Figure 6 shows the variation in the spectrum of the hydrosol 3 days later. It can be seen that the LSPR band decreased by about 10% in intensity, was broadened, and shifted by about 5 nm to longer wavelengths (red shift). The intensity and position of the IBM band remained unchanged, which indicated that the number of silver atoms in the hydrosol was retained. The hydrosol was then exposed to UV light (~20 s). The absorption of the hydrosol returned to nearly the initial state. In other words, the LSPR band increased in intensity, narrowed, and shifted to the blue region. The described charging 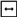 discharging procedure could be repeated. When the hydrosol was stored, nanoparticles lost the excess charge. A decrease in the charge can be caused both by a decrease in the concentration of free electrons and by the partial ionization of adatoms on the surface of a silver particle. The repeated photochemical charging of the hydrosol with electrons from the donor (${\mathrm{CO}}_{2}^{-\cdot }$ radical ion) restored the nanoparticle charge, which resulted in the restoration of the former position and shape of the LSPR band. In these experiments, we used a hydrosol with an invariable composition of silver nanoparticles (size and size distribution).

According to the Mie–Drude theory [16,17,18], the wavelength corresponding to plasmon resonance (λ_m_) is given by the following relation:

(4) $$\lambda_{m}^{2}=\lambda_{c}^{2}\;\left(e_{0}+2n_{0}^{2}\right)$$

where ε_0_ is the high-frequency dielectric constant of the metal, n_0_ is solvent refractive index, and λ_c_ is the electron plasma wavelength in the metal. It follows from this equation that the resonance absorption of light by surface plasmons is attained at the wavelength at which ε_0_ = −2n_0_^2^. The $\lambda_{c}^{2}$ value is determined from the equation:

(5) $$\lambda_{c}^{2}=\frac{{\left(2pc\right)}^{2}m}{4{\mathrm{pN}}_{e}e^{2}}$$

Here, m is the effective mass of an electron, N_e_ is the density of free electrons in the metal, *c* and *e* are the speed of light and the charge of an electron, respectively. Equations (4) and (5) show that increase in the concentration of free electrons in the particle N_e_ should induce a blue shift of the adsorption band, as is actually observed in the experiments (Figure 2a). Unfortunately, in the framework of this theory, it is impossible to calculate the concentration of electrons in a nanoparticle from the shift ∆λ_max_, because of the uncertainty of many nanoparticle parameters. However, the following expression relating the initial and final positions of bands ($\lambda_{\max}^{i}$ and $\lambda_{\max}^{f}$) to the relative concentrations of electrons in the metal core ($n_{e}^{i}$ and $n_{e}^{f}$) can be derived from Equation (5):

(6) $$\frac{n_{e}^{f}}{n_{e}^{i}}={\left(\frac{\lambda_{\max}^{i}}{\lambda_{\max}^{f}}\right)}^{2}$$

Equation (6) makes it possible to estimate the change in the relative concentration of electrons in the metal from the shift of the LSPR band. In order to derive an analytic correlation, we will transform Equation (6). It will be assumed that $n_{e\;}^{f}{=n}_{e\;}^{i}+\Delta n_{e}$ and $\lambda_{\max}^{i}{=\lambda }_{\max}^{f}+\Delta \lambda$. Since the $\frac{{\Delta \lambda }^{2}}{\lambda_{\max}^{i}{}^{2}}$ value is small (≤2%), expression (6) can be transformed into the simple equation

(7) $$\Delta n_{e}\approx \;\frac{2n_{e}^{i}}{\lambda_{\max}^{f}}\Delta \lambda$$

which establishes the direct dependence of the change in the relative electron concentration in the nanoparticle ∆n_e_ on the shift of the LSPR band (∆λ). The greater the blue shift, the greater the concentration of electrons in the metal.

**Figure 7:**
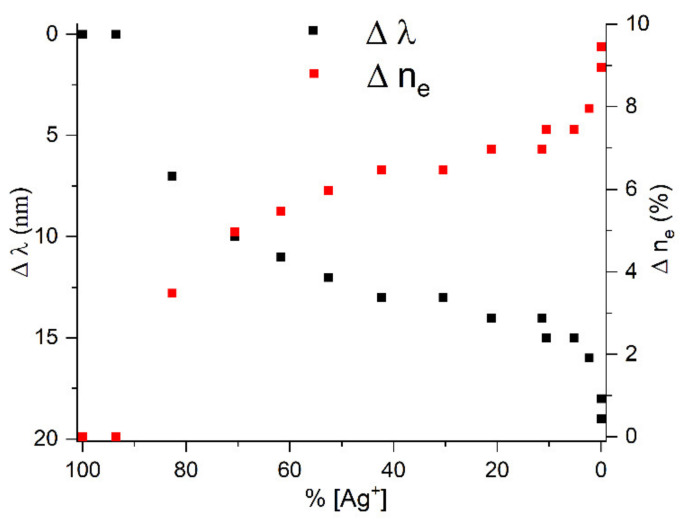
Variation in ∆λ and ∆n_e_ in silver nanoparticles vs. Ag^+^ ion concentration.

Figure 7 shows the dependences of the LSPR shift (∆λ) and the relative electron concentration in the nanoparticle (∆n_e_) calculated from Equation (7) on the concentration of Ag^+^ ions. The relative concentration of [Ag^+^] ions was determined from the intensity of the IBT band (the intensity of the band with completely reduced silver ions was taken as 100%). It can be seen that after the completion of Ag^+^ reduction, the LSPR band continues to shift to shorter wavelengths under UV irradiation. This is strong evidence that the decrease in the silver ion concentration is not the only cause for the shift of the LSPR band, and after complete reduction the electron concentration in the metal increases by another mechanism.

**Figure 8:**
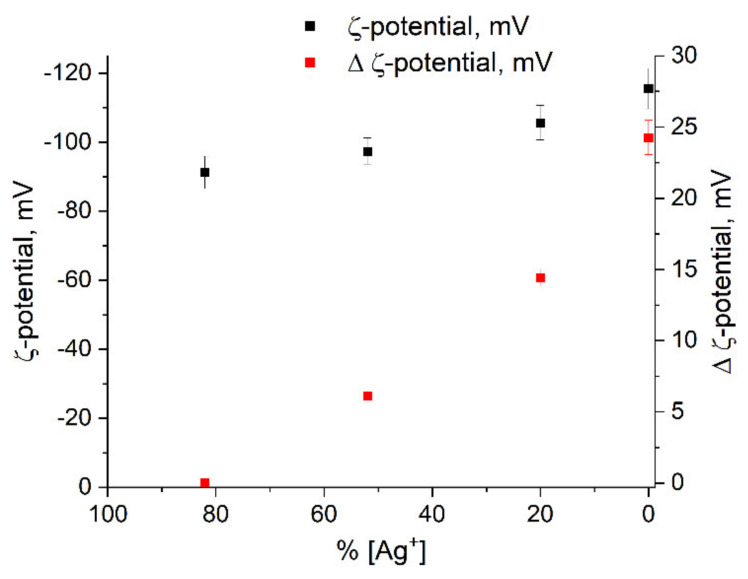
Variation in ζ-potential of silver colloid vs. Ag^+^ ion concentration.

The ζ-potential of silver colloids was determined by dynamic light scattering (DLS). The potential has a negative sign. This indicates that the potential determining layer of the colloid is formed from anions (apparently,${\;\mathrm{HCO}}_{3}^{-}$). The nanoparticle forms a colloidal particle together with the counterions of the dense adsorption layer of DEL (Ag^+^ ions and other cations). The colloidal particle is surrounded by counterions from the diffuse part. The ζ-potential is the potential at the boundary between a colloidal particle capable of moving in an electric field and the surrounding liquid—i.e., the potential of the sliding surface of a particle in a colloidal solution. The large absolute value of the potential (about −100 mV) indicates the high stability of the hydrosol. There is a clear tendency towards an increase in the potential in absolute value with a decrease in the concentration of free Ag^+^ ions in solution, as a result of their reduction and the formation of metal nanoparticles (Figure 8). The same figure shows the change in Δζ = ζ_n −_ ζ_1_, where x_1_ is the potential at the first stage of the formation of nanoparticles and ζ_n_ are the potentials at the subsequent stages. We observe a distinct increase in potential during the formation of the hydrosol.

The ζ-potential change correlates with the change in ∆n_e_ (compare Figure 7 and Figure 8). It is known [40] that the ζ-potential is determined by the thickness of the diffuse layer of the colloid. The greater the thickness, the greater the value of the ζ-potential. An increase in the density of electrons in the metal nuclei of the colloid should decrease the concentration of potential determining ions in the adsorption layer. In turn, this should be accompanied by an increase in the concentration in the diffuse layer. As a result, the thickness of the diffuse layer increases, which leads to the observed increase in the value of the ζ-potential.

The blue shift of 19 nm is caused by an approximately 10% increase in the free electron density. Previously [21], the LSPR band was also found to be blue shifted during the photochemical reduction of 1.1 × 10^−4^ mol L^−1^ Ag^+^ ions in a solution containing 1.0 mol L^−1^ of propanol and 2 × 10^−2^ mol L^−1^ of acetone, and also 2 × 10^−4^ mol L^−1^ of polyethyleneimine as a stabilizer. We analyzed the experimental data presented in the cited study and found that the band shifted during the reduction from the initial position of approximately 402 nm to 383 nm by completion of the process. The use of the calculation method proposed in the present paper demonstrates that the electron density in the nanoparticles increases during this process by 9.4%. Note also that, according to the Mie–Drude theory [16,17,18,19], the increase in the electron concentration in the metal brings about not only a blue shift of the LSPR absorption band, but also a decrease in the width of this band. The full width at half-height of the absorption band (ω) is determined by the following expression:

w = (ε_0_ + 2n_0_^2^)c/2s, (8)

After substitution of the electrical conductivity s = N_e_e^2^R/mu_F_ (m is the effective electron mass, u_F_ is electron velocity at the Fermi level, and R is the particle radius) to the denominator of the preceding equation, it becomes clear that the width of the LSPR band should decrease with the increasing density of free electrons N_e_.

As can be seen (Figure 2b), this is observed experimentally. The Ag^+^ ions on the nanoparticle surface behave as acceptors withdrawing the conduction electrons. During the photochemical reduction, their concentration in the solution decreases, which is accompanied by a decrease in the concentration of ions ${\mathrm{Ag}}_{\mathrm{ad}}^{+}$ due to the shift of the equilibrium on Equation (9) to the right:

(9) $${\mathrm{Ag}}_{n}\cdot {\mathrm{mAg}}_{\mathrm{ad}}^{+}↔\;{\mathrm{Ag}}_{n}\cdot \left(m-x\right){\mathrm{Ag}}_{\mathrm{ad}}^{+}+{\;\mathrm{xAg}}^{+}$$

Thus, as the concentration of Ag^+^ ions on silver nanoparticles decreases, the concentration of conduction electrons in the nanoparticles increases. Subsequently, after complete reduction in Ag^+^ ions in the solution volume and formation of metal nanoparticles (stationary IBT absorption, Figure 3, curve 1), the concentration of electrons in the nanoparticles continues to increase (increasing LSPR absorption, Figure 3, curve 2). Apparently, this is caused by the transfer of additional electrons to the silver nanoparticle from the formed donors (radical ions ${\mathrm{CO}}_{2}^{-\cdot }$):

(10) $${\mathrm{Ag}}_{n}+\;{\mathrm{wCO}}_{2}^{-\cdot }\;\to \;{\mathrm{Ag}}_{n}^{w-}+\;{\mathrm{wCO}}_{2}$$

During the formation of silver nanoparticles, the structure of the DEL changes due to a change in the solution composition: the disappearance of Ag^+^ ions from the surface of the nanoparticles and the replacement of oxalate ions by the formed carbonate ions. As a result, the electrostatic stabilization of a nanoparticle is realized.

The detected effect of the electron density evolution of silver nanoparticles during their redox formation may also be common for other metals. Notably, in the process of gold [41] and platinum [42] hydrosols synthesis, shift of optical spectra bands’ maximum, sensitive to surface conditions, was observed. Increasing the electron density on the surface of metal particles increases their catalytic activity [43,44]. Accordingly, the electronic charging and discharging of nanoparticles affects the efficiency of various catalytic reactions involving metals in the nanoscale state [45,46].

## 3. Materials and Methods

### 3.1. Chemicals and Materials

Silver perchlorate monohydrate (AgClO_4_ ∙ H_2_O, 99%, Alfa Aesar, Ward Hill, MA, USA) and potassium oxalate (K_2_C_2_O_4_, special purity grade, 99.9%, Reakhim, Moscow, Russia) were used. Double-distilled water was used as a solvent.

### 3.2. Synthesis Procedure

The “pure” silver hydrosol containing silver nanoparticles (NPs) and stabilizing carbonate ions was prepared by a reduction in silver ions by oxalate ions under the action of pulsed UV radiation (28). First, the solution was deaerated by evacuation. Irradiation was carried out in a special glass vessel equipped with a quartz cell (2–4 mL volume) with an optical path of 5 or 10 mm. The solutions were irradiated with a pulsed xenon lamp at the total radiation flux intensity I_UV_ = 6.0 × 10^20^ quanta per second. The light flux from the xenon lamp covers the whole UV and visible regions and is most similar to the solar light emission.

### 3.3. Instrumentation

The optical spectra were measured with a Cary 100 Scan spectrophotometer (Varian Inc., Palo Alto, CA, USA) equipped with a Peltier thermostatic cell at 20 °C. The hydrodynamic size (d) and the ζ-potential of silver nanoparticles were determined by dynamic light scattering on a Delsa Nano C instrument (Beckman Coulter Inc., Brea, CA, USA). The wavelength of the scattered laser radiation was λ = 658 nm. The nanoparticle size and polydispersity were determined using a JEM-2100 transmission electron microscope (TEM) (JEOL, Akishima, Tokyo, Japan) operating at an accelerating voltage of 200 kV.

## 4. Conclusions

The results of our study show that the spectrophotometric method is effective for studying changes in the electronic state and properties of silver nanoparticles in aqueous solutions, as well as for determining the mechanism of metal nucleation in a homogeneous medium. It was shown that the electronic state of nanoparticles is changed during their formation. This fact is explained by an increase in the concentration of electrons on the surface as a result of the reduction in adsorbed Ag^+^ ions and electron injection by organic radicals. As a result of that, the structure of the electrical double layer also changes due to a change in the surface of silver nanoparticles.

## References

[1] Liu L., Corma A. (2018). Metal Catalysts for Heterogeneous Catalysis: From Single Atoms to Nanoclusters and Nanoparticles. Chem. Rev..

[2] Zhang N., Chen F., Guo L. (2019). Catalytic Activity of Palladium-doped Silver Dilute Nanoalloys for Formate Oxidation from a Theoretical Perspective. Phys. Chem. Chem. Phys..

[3] Jin R., Zeng C., Zhou M., Chen Y. (2016). Atomically Precise Colloidal Metal Nanoclusters and Nanoparticles: Fundamentals and Opportunities. Chem. Rev..

[4] Pan Z.Y., Zhou J., Zou H.Z., Li Y.F., Gao P.F., Huang C.Z. (2021). In situ investigating the size-dependent scattering signatures and sensing sensitivity of single silver nanocube through a multi-model approach. J. Colloid Interface Sci..

[5] Zhang H., Jin M., Xiong Y., Lim B., Xia Y. (2013). Shape-Controlled Synthesis of Pd Nanocrystals and Their Catalytic Applications. Acc. Chem. Res..

[6] Song J., Guan R., Liu X., Jiang C., Hou G. (2018). Tuning Local Surface Plasmon Resonance of Silver and Photoluminescence Intensity Enhancement by Adding Copper. J. Phys. Chem. Solids.

[7] Shalabney A., Abdulhalim I. (2011). Sensitivity-Enhancement Methods for Surface Plasmon Sensors. Laser Photonics Rev..

[8] Zhang L., Wang E. (2014). Metal Nanoclusters: New Fluorescent Probes for Sensors and Bioimaging. Nano Today.

[9] Tao Y., Li M., Ren J., Qu X. (2015). Metal Nanoclusters: Novel Probes for Diagnostic and Therapeutic Applications. Chem. Soc. Rev..

[10] Webb J., Bardhan R. (2014). Emerging Advances in Nanomedicine with Engineered Gold Nanostructures. Nanoscale.

[11] Somlyai-Sipos L., Baumli P., Sycheva A., Kaptay G., Szőri-Dorogházi E., Kristály F., Mikó T., Janovszky D. (2020). Development of Ag Nanoparticles on the Surface of Ti Powders by Chemical Reduction Method and Investigation of Their Antibacterial Properties. Appl. Surf. Sci..

[12] Hassanzadeh P. (2020). Nanotheranostics against COVID-19: From Multivalent to Immune-Targeted Materials. J. Control Release.

[13] Jadhav S.A., Biji P., Panthalingal M.K., Murali C., Kulkarni A., Rajkumar S., Joshi D.S., Natarajan S. (2021). Development of Integrated Microfluidic Platform Coupled with Surface-Enhanced Raman Spectroscopy for Diagnosis of COVID-19. Med. Hypotheses.

[14] Teengam P., Siangproh W., Tuantranont A., Vilaivan T., Chailapakul O., Henry C.S. (2017). Multiplex Paper-Based Colorimetric DNA Sensor Using Pyrrolidinyl Peptide Nucleic Acid-Induced AgNPs Aggregation for Detecting MERS-CoV, MTB, and HPV Oligonucleotides. Anal. Chem..

[15] Mie G. (1908). Beiträge zur Optik trüber Medien, speziell kolloidaler Metallösungen. Ann. Phys..

[16] Van de Hulst H.C. (1957). Light Scattering by Small Particles.

[17] Kerker M. (1969). The Scattering of Light and Other Electromagnetic Radiation.

[18] Kreibig U., Vollmer M. (1995). Optical Properties of Metal Clusters.

[19] Qu Y., Cheng R., Su Q., Duan X. (2011). Plasmonic Enhancements of Photocatalytic Activity of Pt/n-Si/Ag Photodiodes Using Au/Ag Core/Shell Nanorods. J. Am. Chem. Soc..

[20] Herne T.M., Ahern A.M., Garrell R.L. (1991). Surface-Enhanced Raman Spectroscopy of Peptides: Preferential N-Terminal Adsorption on Colloidal Silver. J. Am. Chem. Soc..

[21] Henglein A. (1998). Colloidal Silver Nanoparticles: Photochemical Preparation and Interaction with O_2_, CCl_4_, and Some Metal Ions. Chem. Mater..

[22] Ershov B.G., Abkhalimov E.V., Roldughin V.I., Rudoy V.M., Dement’eva O.V., Solovov R.D. (2015). Adsorption of Ozone and Plasmonic Properties of Gold Hydrosol: The Effect of the Nanoparticle Size. Phys. Chem. Chem. Phys..

[23] Muhammed M.A.H., Döblinger M., Rodríguez-Fernández J. (2015). Switching Plasmons: Gold Nanorod-Copper Chalcogenide Core-Shell Nanoparticle Clusters with Selectable Metal/Semiconductor NIR Plasmon Resonances. J. Am. Chem. Soc..

[24] Yuan K., Qin R., Yu J., Li X., Li L., Yang X., Yu X., Lu Z., Zhang X., Liu H. (2020). Effects of Localized Surface Plasmon Resonance of Ag Nanoparticles on Luminescence of Carbon Dots with Blue, Green and Yellow Emission. Appl. Surf. Sci..

[25] Patel M., Pataniya P.M., Late D.J., Sumesh C.K. (2020). Plasmon-Enhanced Photoresponse in Ag-WS_2_/Si Heterojunction. Appl. Surf. Sci..

[26] Celebrano M., Kukura P., Renn A., Sandoghdar V. (2011). Single-molecule imaging by optical absorption. Nat. Photonics.

[27] Ringe E., Sharma B., Henry A.-I., Marks L.D., Van Duyne R. (2013). Single nanoparticle plasmonics. Phys. Chem. Chem. Phys..

[28] Murali G., Vattikuti S.V.P., Kshetri Y.K., Lee H., Modigunta J.K.R., Reddy C.S., Park S., Lee S., Poornaprakash B., Lee H. (2021). Near-infrared-activated Z-scheme NaYF_4_:Yb/Tm@Ag_3_PO_4_/Ag@g-C_3_N_4_ photocatalyst for enhanced H_2_ evolution under simulated solar light irradiation. Chem. Eng. J..

[29] Vattikuti S.V.P., Nagajyothi P.C., Devarayapalli K.C., Yoo K., Nam N.D., Shim J. (2020). Hybrid Ag/MoS2 nanosheets for efficient electrocatalytic oxygen reduction. Appl. Surf. Sci..

[30] Nagajyothi P.C., Reddy L.V., Devarayapalli K.C., Vattikuti S.V.P., Wee Y.J., Shim J. (2021). Environmentally Friendly Synthesis: Photocatalytic Dye Degradation and Bacteria Inactivation Using Ag/f-MWCNTs Composite. J. Clust. Sci..

[31] Abkhalimov E.V., Ershov V.A., Ershov B.G. (2019). “Pure” Silver Hydrosol: Nanoparticles and Stabilizing Carbonate Ions. J. Nanopart. Res..

[32] Kai T., Zhou M., Johnson S., Ahn H.S., Bard A.J. (2018). Direct Observation of C_2_O_4_^•−^ and CO_2_^•−^ by Oxidation of Oxalate within Nanogap of Scanning Electrochemical Microscope. J. Am. Chem. Soc..

[33] Anderson G.K., Lumetta G.J., Siria J.W. (1992). Photochemical Reactions of Diphosphineplatinum(II) Oxalate Complexes. J. Organomet. Chem..

[34] Wardman P. (1989). Reduction potentials of one-electron couples involving free radicals in aqueous solution. J. Phys. Chem. Ref. Data.

[35] Henglein A. (1990). Remarks on the electrochemical potential of small silver clusters in aqueous solution. Ber. Bunsenges. Phys. Chem..

[36] Gupta S., Prakash R. (2014). Photochemically Assisted Formation of Silver Nanoparticles by Dithizone, and Its Application in Amperometric Sensing of Cefotaxime. J. Mater. Chem. C.

[37] Ershov B.G., Janata E., Henglein A., Fojtic A. (1993). Silver Atoms and Clusters in Aqueous Solution: Absorption Spectra and The Particles Growth in Absence of Stabilizing Ag^+^ Ions. J. Phys. Chem..

[38] Ershov B.G. (1999). Short-Lived Metal Clusters in Aqueous Solutions: Formation, Identification, and Properties. Russ. Chem. Bull..

[39] Belloni J., Marignier J.-L., Mostafavi M. (2020). Mechanisms of Metal Nanoparticles Nucleation and Growth Studied by Radiolysis. Radiat. Phys. Chem..

[40] Russel W.B., Saville D.A., Schowalter W.R. (1989). Colloidal Dispersions.

[41] Sánchez-Iglesias A., Claes N., Solís D.M., Taboada J.M., Bals S., Liz-Marzán L.M., Grzelczak M. (2018). Reversible Clustering of Gold Nanoparticles under Confinement. Angew. Chem. Int. Ed. Agew. Chem..

[42] Chen C.-W., Takezako T., Yamamoto K., Serizawa T., Akashi M. (2000). Poly(N-vinylisobutyramide)-Stabilized Platinum Nanoparticles: Synthesis and Temperature-Responsive Behavior in Aqueous Solution. Colloids. Surf. A Physicochem. Eng. Asp..

[43] Mavroyannis C. (1963). The interaction of neutral molecules with dielectric surfaces. Mol. Phys..

[44] Yagodovskii V.D. (2015). Modifying the Catalytic and Adsorption Properties of Metals and Oxides. Russ. J. Phys. Chem. A.

[45] Von Vol’kenshtein F.F. (1964). The electronic theory of catalysis on semiconductors. Angew. Chem..

[46] Liao G., Fang J., Li Q., Li S., Xu Z., Fang B. (2019). Ag-Based Nanocomposites: Synthesis and Applications in Catalysis. Nanoscale.

